# Supplementary material for: Genome-Wide Identification and Characterization of MYB Transcription Factors in Sudan Grass under Drought Stress
Source: Plants (Basel). 2024 Sep 21;13(18):2645. doi: 10.3390/plants13182645 (PMC11435211; doi:10.3390/plants13182645)
Supplement: Supplementary file 1 [file plants-13-02645-s001.zip › Figure S1.pdf]

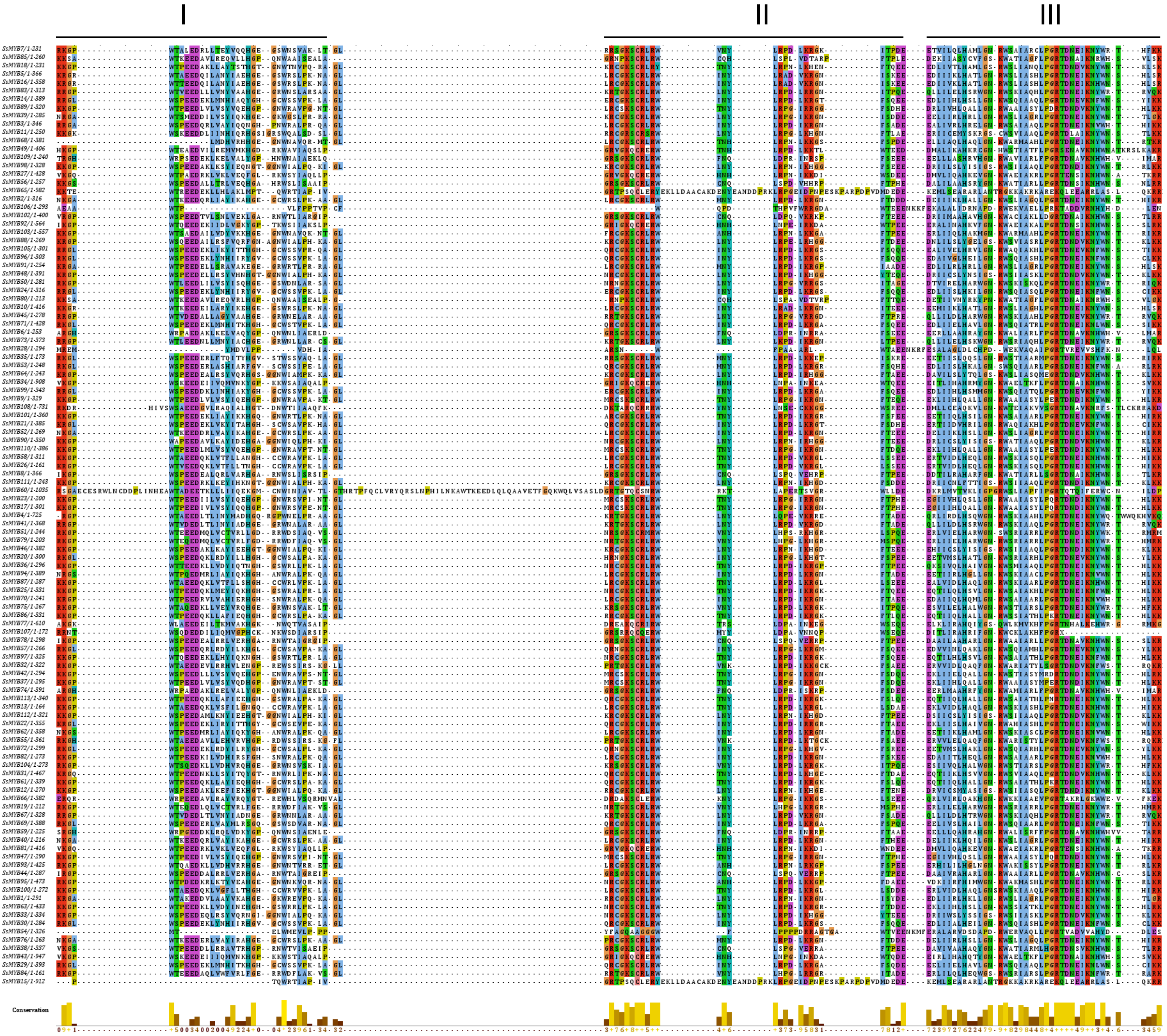
I
II
III
Conservation
