# Supplementary figures and images for: Genome-Wide Identification and Characterization of MYB Transcription Factors in Sudan Grass under Drought Stress
Source: Plants (Basel). 2024 Sep 21;13(18):2645. doi: 10.3390/plants13182645 (PMC11435211; doi:10.3390/plants13182645)

Motif 1

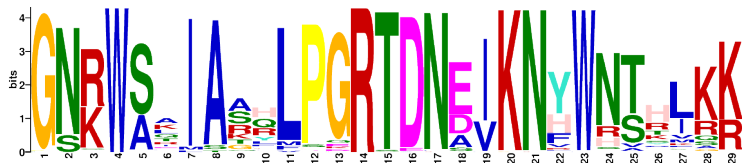

Motif 2

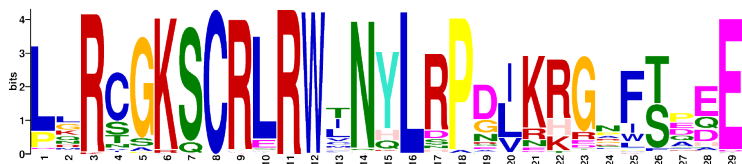

Motif 3

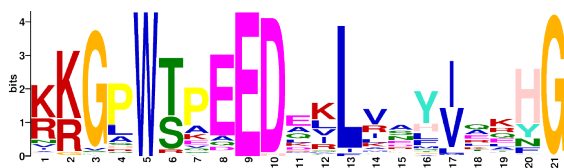

Motif 4

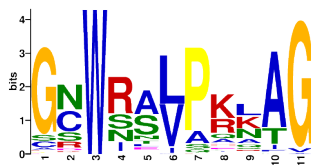

Motif 5

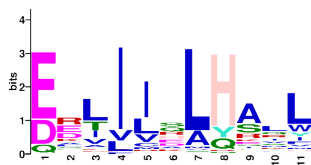

Motif 6

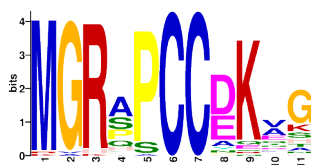

Motif 7

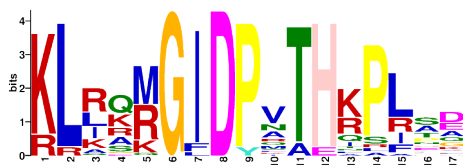

Motif 8

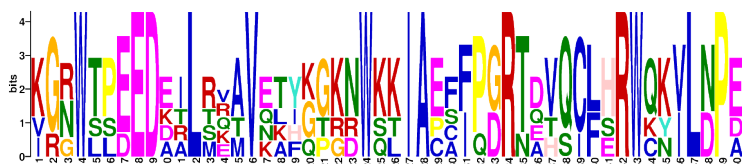

Motif 9

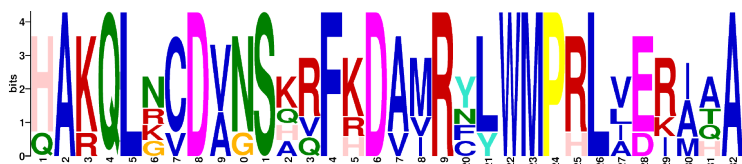

Motif 10

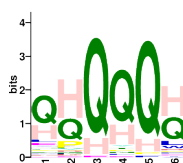

Supplement: Supplementary file 1 [file plants-13-02645-s001.zip › Figure S2.pdf]

# Top 25 of KEGG Enrichment

Pathway

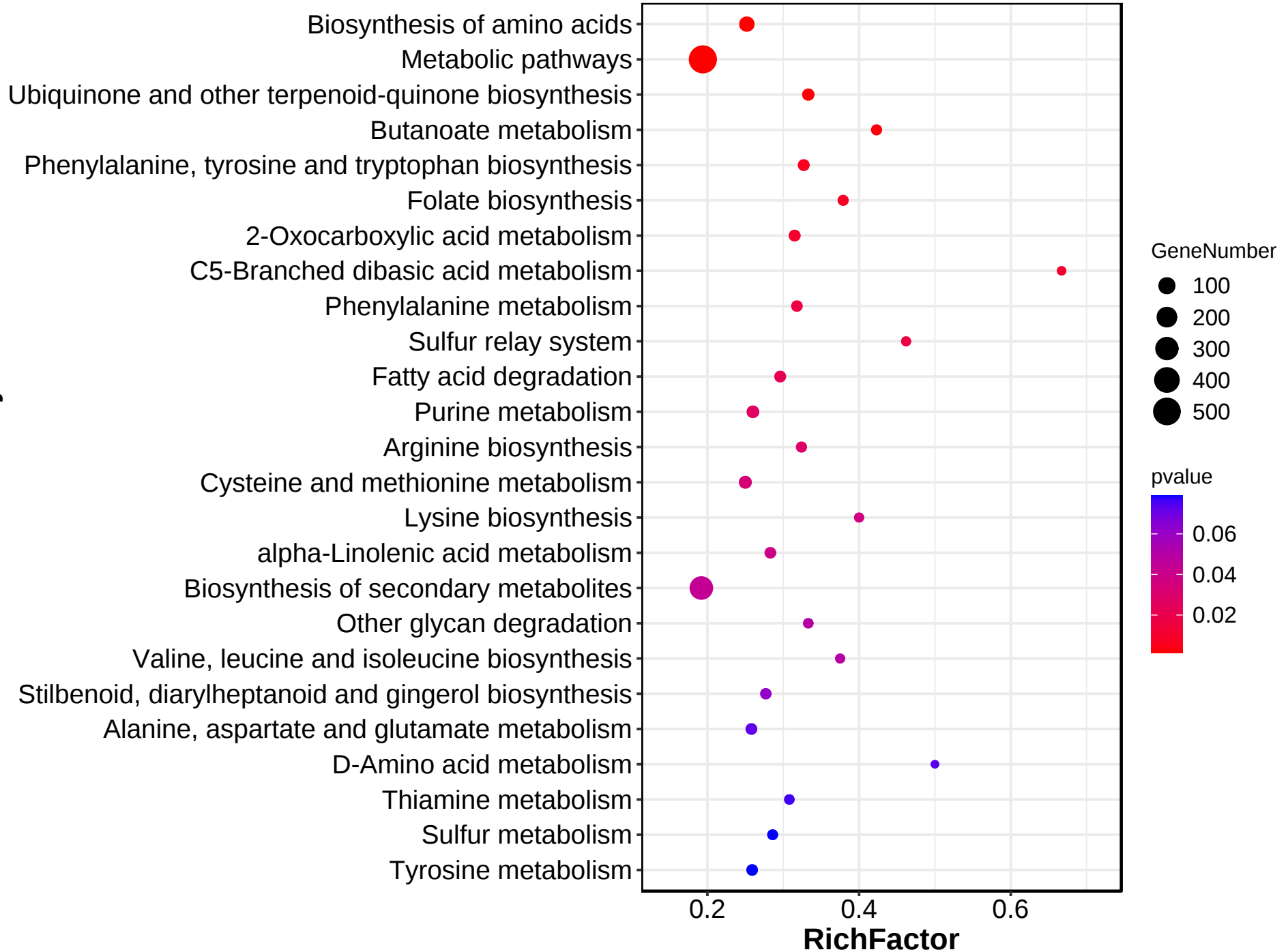

Supplement: Supplementary file 1 [file plants-13-02645-s001.zip › Figure S3.pdf]
